# Supplementary material for: MRTF-dependent cytoskeletal dynamics drive efficient cell cycle progression
Source: J Cell Sci. 2025 Dec 29;138(24):jcs264444. doi: 10.1242/jcs.264444 (PMC12813672; doi:10.1242/jcs.264444)
Supplement: Supplementary information [file joces-138-264444-s1.pdf]

A

Marker	Expression relative to WT, ± SEM	Expts (n)
p16	0.75 ± 0.09	(3)
p21	0.80 ± 0.39	(4)
p27	5.91 ± 1.05	(4)
p57	2.13 ± 0.56	(4)
CDK1	0.73 ± 0.09	(5)
CKS2	0.61 ± 0.06	(5)
CDK4	1.79 ± 0.52	(3)
CycD	0.72 ± 0.11	(2)
TLR2	6.43 ± 1.89	(3)
p53	0.38 ± 0.03	(3)
pS15-p53	1.11 ± 0.22	(3)

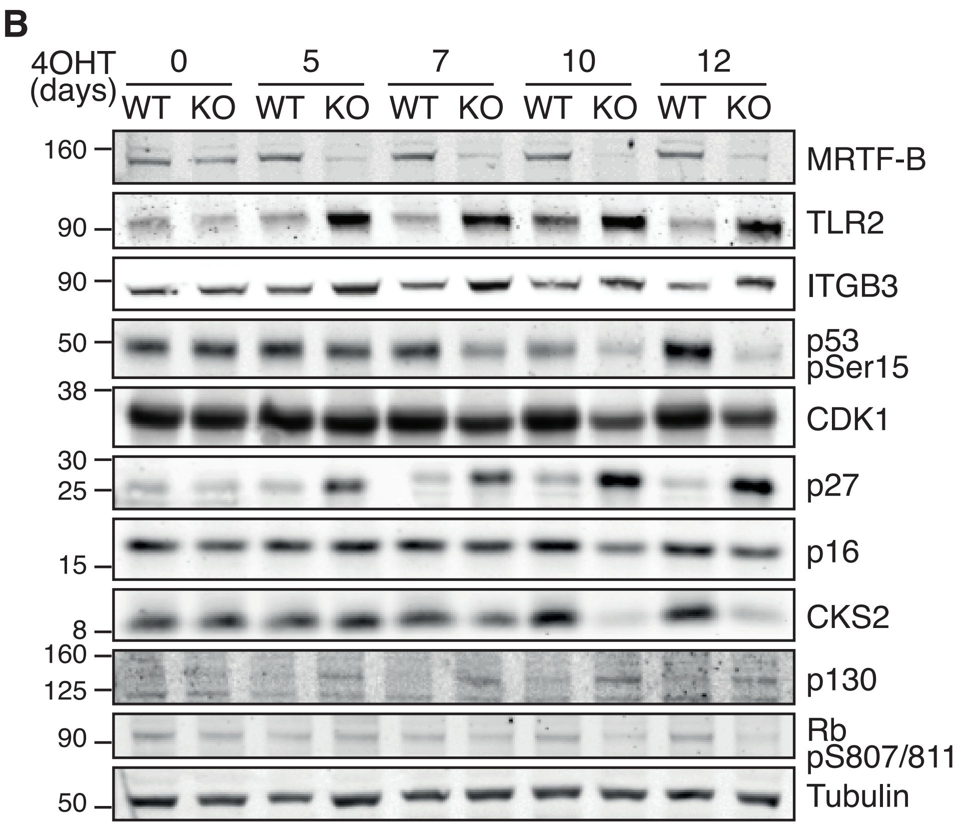

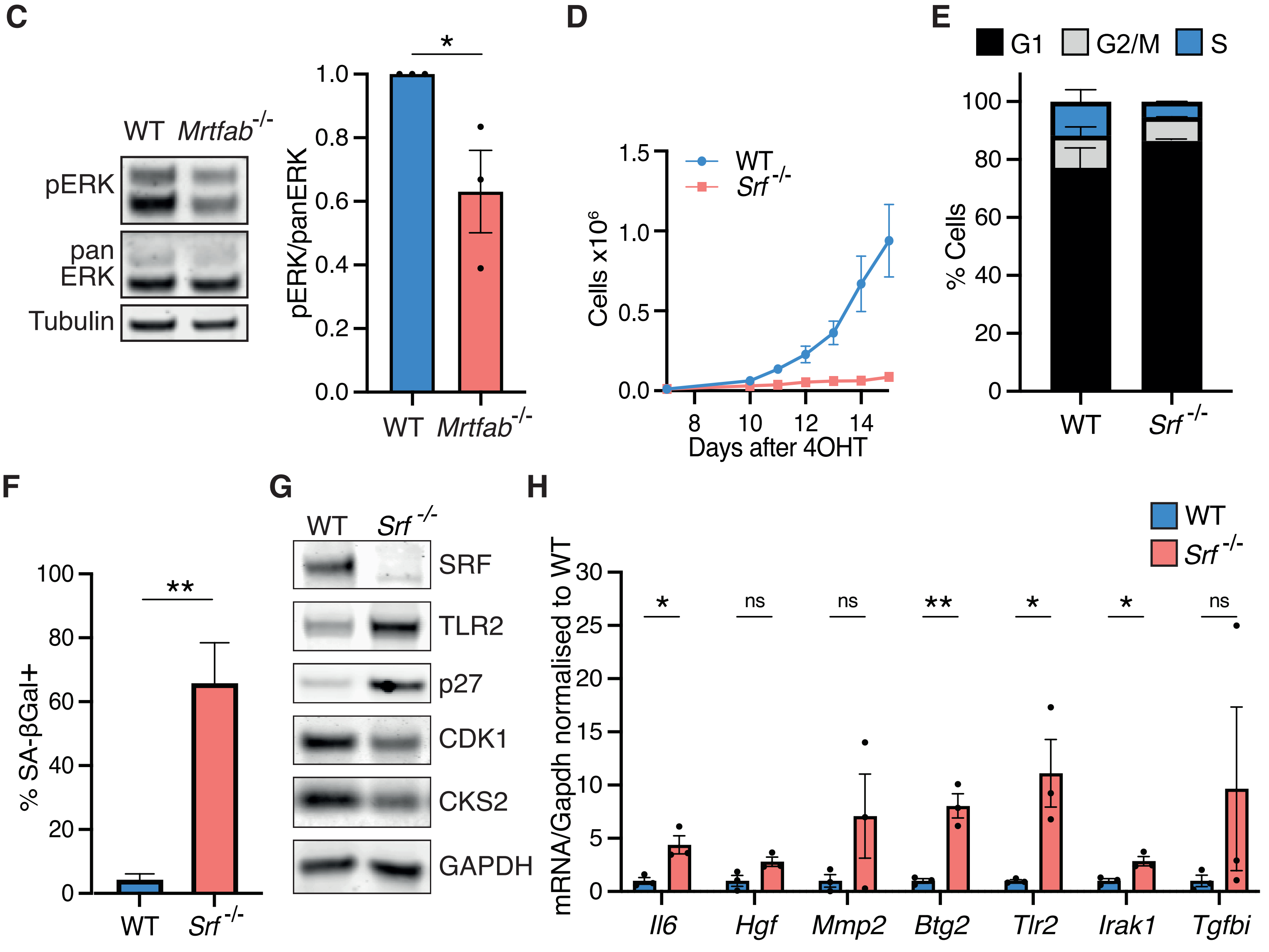

Journal of Cell Science • Supplementary information

Fig. S1. Defective proliferation of MRTF- or SRF-null MEFs

A) Summary of cell cycle and senescence marker expression. Results are from five independent analyses, all of which included 3 WT and 3 *Mrtfab*$^{-/-}$ pools (note that not all markers were evaluated in each analysis). For each experiment, marker expression levels in each *Mrtfab*$^{-/-}$ pool were normalised to its mean expression level in the WT pools. Data is mean marker expression in the 3 *Mrtfab*$^{-/-}$ pools ± SEM (± half-range for CycD).

B) Immunoblot analysis of cell cycle and senescence markers in each pool of WT or *Mrtfab*$^{-/-}$ MEFs at day 12 after 4OHT.Immunoblotting of WT (pool 80) and *Mrtfab*$^{-/-}$ (pool 86) MEFs at the indicated times after 4OHT. Spearman correlation between relative levels of the markers TLR2, p53, CDK1, p27, p16, and CKS2 were as follows: WT vs *Mrtfab*$^{-/-}$, untreated, 0.94; WT vs *Mrtfab*$^{-/-}$, 12 days 4OHT, 0.26; WT, untreated vs WT 12 days 4OHT, 0.89. Similar results were obtained with the other pools.

C) ERK activity in day 12 WT and *Mrtfab*$^{-/-}$ MEFs. Left, representative immunoblot of pERK and panERK (pools 80 and 86). Right, quantification showing mean of the three independent pools of each genotype. Results are normalised to WT. Statistical analysis, unpaired t-test. *, $p<0.05$.

D) Pools of MEFs derived from WT or *Srf*$^{-/-}$ MEFs (three pools per genotype, each derived from one embryo) were treated with 4OHT and growth curves determined as in Figure 1A. For each pool, cell counts at each time point are the mean of three technical replicates. Data points are the mean cell counts of the three independent cell pools ± SEM. A representative of three independent experiments is shown.

E) Cell cycle distribution of WT or *Srf*$^{-/-}$ MEFs on day 12 after 4OHT evaluated by PI staining. Representative of two independent experiments.

F) SA-βgal staining of WT and *Srf*$^{-/-}$ MEFs on day 12 after 4OHT. Data are mean of the three independent pools (>100 cells scored per pool) ± SEM. Statistical analysis, unpaired t-test. **, $p<0.01$.

G) Immunoblotting of WT or *Srf*$^{-/-}$ MEFs on day 12 after 4OHT. Data are from pools MEF4 and Bis1. A representative of three independent experiments is shown; similar results were obtained with the other pools.

H) RT-qPCR of mRNA levels of SASP factors in day 12 WT or *Mrtfab*$^{-/-}$ MEFs after 4OHT. Data are mean of the three independent pools ± SEM; significance, unpaired t-test. ns, not significant; *, $p<0.05$; **, $p<0.01$.

Journal of Cell Science • Supplementary information

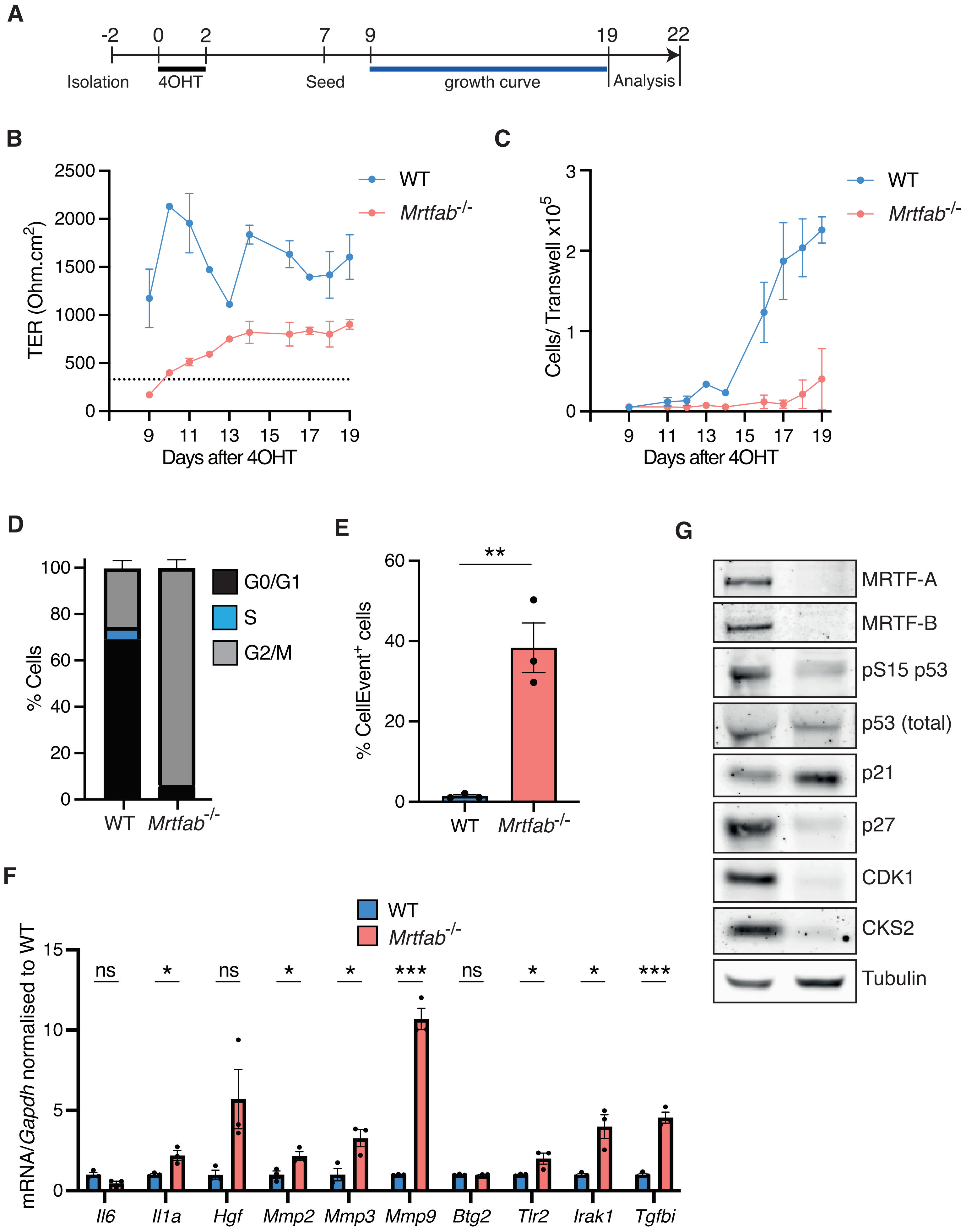

Journal of Cell Science • Supplementary information

Fig. S2. Defective proliferation of MRTF-null primary tracheal epithelial cells

A) Experimental protocol. Mouse tracheal epithelial progenitor cells were isolated, treated with 4OHT, and seeded on collagen/fibronectin coated transwell inserts (10,000 cells per insert).

B) Epithelial barrier function, evaluated by measurement of transepithelial resistance (TER). Data are mean of three independent experiments ± SEM. For the different experiments 4, 3-10 or 6-21 technical replicates were quantified. Dotted line, resistance of confluent cell sheet.

C) Cells from (B) were trypsinised and counted over the course of 12 days.

D) Cell cycle distribution analysis of day 20 BrdU-labelled / PI-stained epithelial cells. Data is from a single experiment with 3 technical replicates ± SEM.

E) Proportion of Cell Event Senescence Green positive epithelial progenitor cells as measured by flow cytometry. Data are mean of three independent experiments on days 19 to 22 after 4OHT, ± SEM. Significance, unpaired t-test; $p<0.01$.

F) RT-qPCR of mRNA levels of SASP factors, normalised to WT cells on day 21 after 4OHT. Data is from a single experiment with 3 technical replicates, ± SEM. Significance, multiple unpaired t-test; ns, not significant; *, $p<0.05$; ***, $p<0.001$. A representative of three independent experiments is shown.

G) Immunoblotting of epithelial progenitor cells on day 19 after 4OHT treatment. Representative of three independent experiments.

Journal of Cell Science • Supplementary information

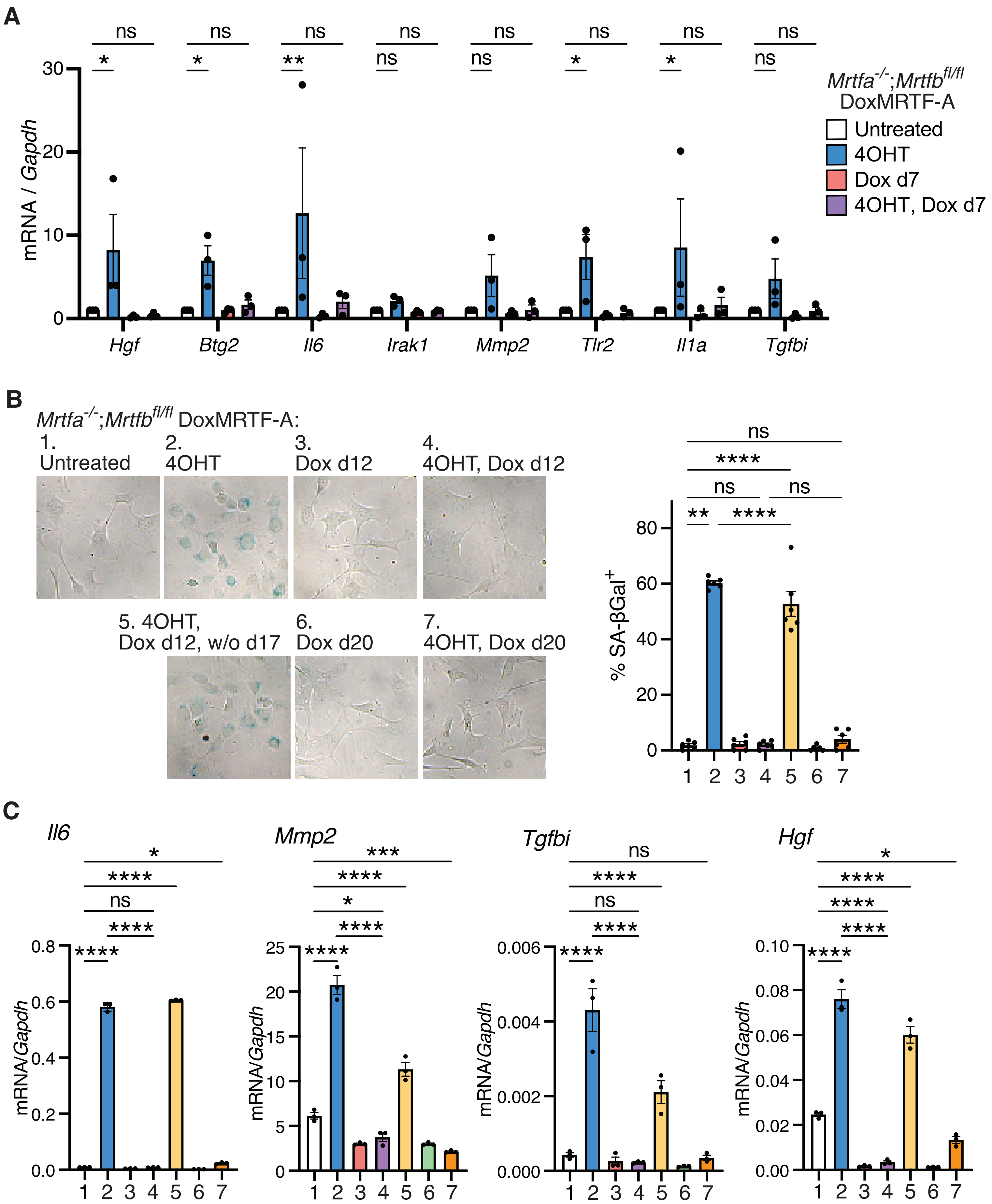

Journal of Cell Science • Supplementary information

Fig. S3. MRTF-A re-expression reverses the proliferative and cytoskeletal defects of *Mrtfab*$^{-/-}$ MEFs.

A) RT-qPCR of SASP markers at day 12 in the three independent *Mrtfab*$^{-/-}$ DoxMRTF-A cell lines treated as in Figure 3A. Data are mean of the three independent lines ± SEM, normalised to untreated cells. Statistical analysis, two-way ANOVA with correction for multiple comparisons. ns, not significant; *, $p<0.05$; **, $p<0.01$.

B) Left, representative images of SA-βGal staining at day 25 in *Mrtfab*$^{-/-}$ DoxMRTF-A cells 1F2 treated as in Figure 3D. Right, quantification; conditions as at left. Data are mean of 6 technical replicates ± SEM. Statistical analysis one-way ANOVA with correction for multiple comparisons; ns, not significant; **, $p<0.01$; ****, $p<0.0001$. Similar results were observed with the 1F5 clone.

C) RT-qPCR of SASP markers at day 25 in *Mrtfab*$^{-/-}$ DoxMRTF-A 1F2 cells treated as in (B). Data are mean of technical replicates ± SEM. Similar results were obtained with 1F5 cells. Statistical analysis, two-way ANOVA with correction for multiple comparisons; ns, not significant; *, $p<0.05$; ***, $p<0.001$; ****, $p<0.0001$.

Journal of Cell Science • Supplementary information

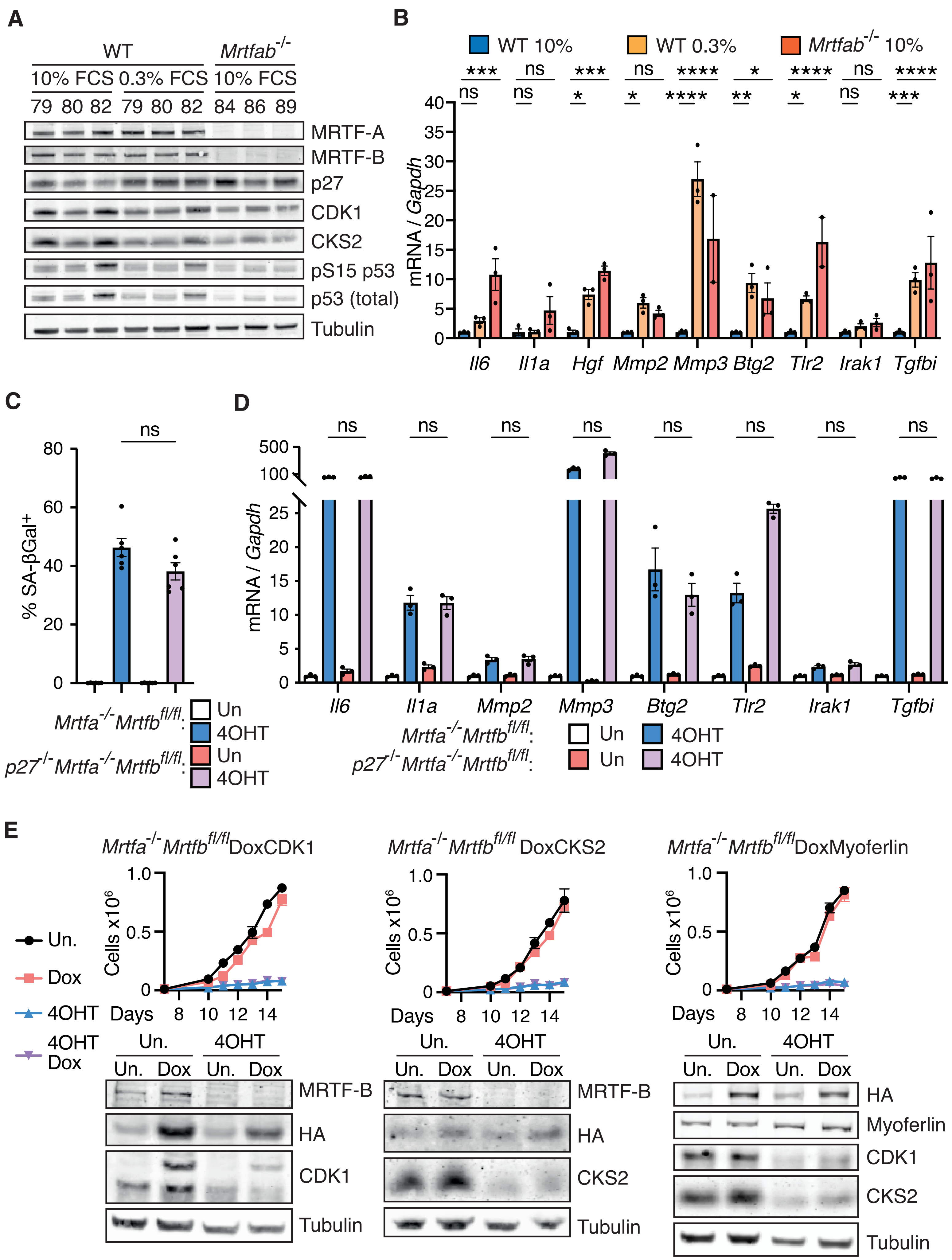

Journal of Cell Science • Supplementary information

Fig. S4. The MRTF-null phenotype exhibits features of quiescence

A) Cells were treated as in Figure 4A but with medium was changed to 0.3% FCS on day 10 *ie* 2 days serum deprivation.

B) RT-qPCR of SASP mRNA levels in cells treated as in Figure 4A. Data are mean ±SEM of three pools of each genotype. Statistical analysis used two-way ANOVA with multiple comparisons using Fisher's LSD test: ns, not significant; *, p<0.05; **, p<0.01; ***, p<0.001;****, p<0.0001.

C) Line 3A2 p27$^{-/-}$*Mrtfa*$^{-/-}$*Mrtfb*$^{fl/fl}$ MEFs treated as in Figure 4F were stained for SA-βGal activity on day 12. Data are mean ± SEM of 6 technical replicates. Statistical analysis was by unpaired t-test: ns, not significant.

D) SASP marker expression in line 3A2 p27$^{-/-}$*Mrtfa*$^{-/-}$*Mrtfb*$^{fl/fl}$ MEFs treated as in Figure 4F were analysed by RT-qPCR on day 12. Data are mean ± SEM of three technical replicates. Statistical analysis was by multiple Mann-Whitney tests. ns, not significant. A representative of two independent experiments is shown.

E) Pool 86 *Mrtfa*$^{-/-}$*Mrtfb*$^{fl/fl}$*Rosa26*$^{Tam\text{-}Cre}$ cells were infected with lentiviruses encoding doxycycline-inducible HA-tagged CDK1 (left), CKS2 (centre) or Myoferlin (right). Following puromycin selection, the resulting cell pools were treated or not with 4OHT and/or doxycycline. Top panels, cell proliferation from days 10 to 15. Data are means of two independent experiments ± SEM. Bottom panels, cell cycle marker expression at day 12.

Journal of Cell Science • Supplementary information

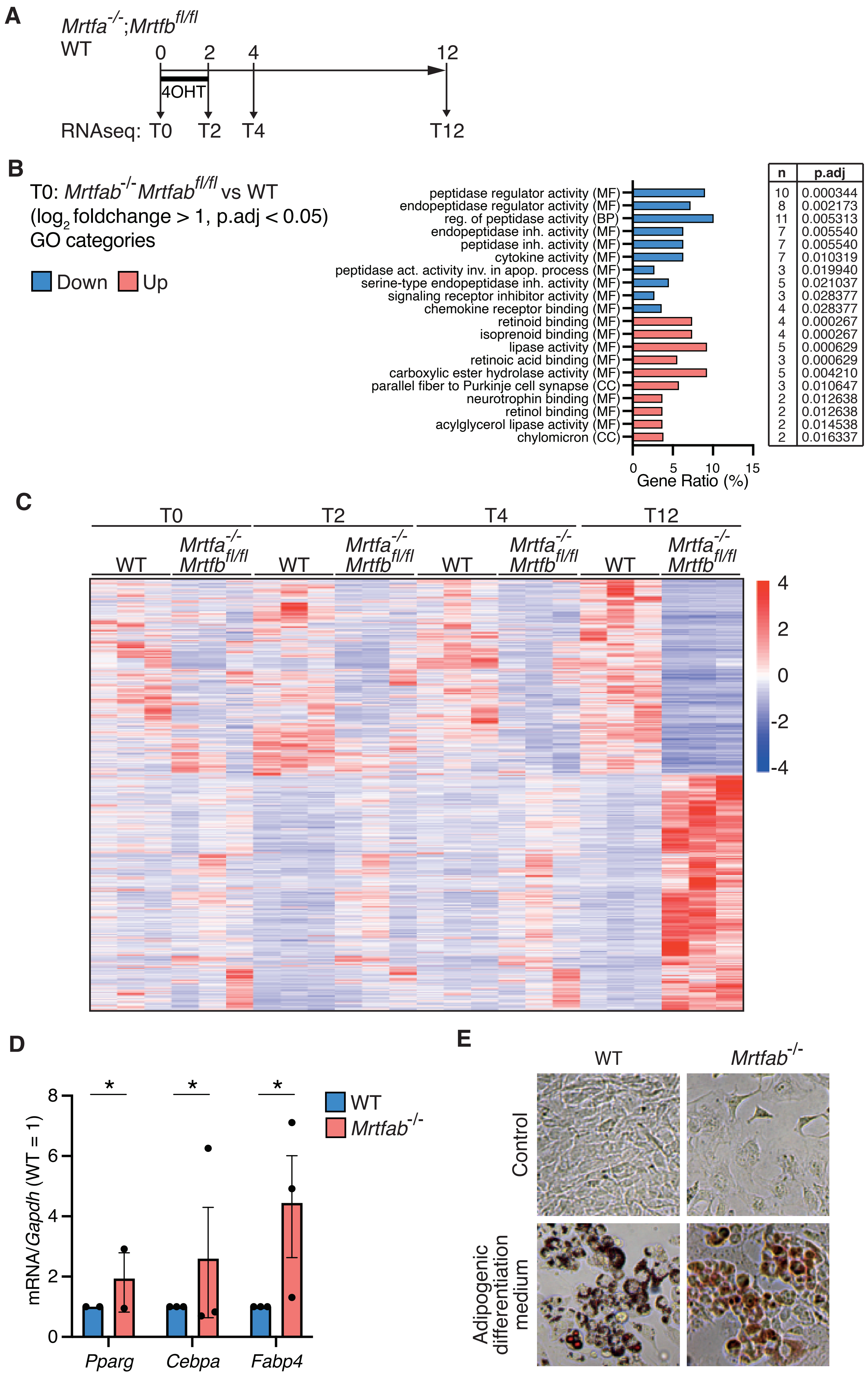

Journal of Cell Science • Supplementary information

Fig. S5. MRTF inactivation results in decreased cytoskeletal gene expression

A) Experimental protocol. WT or *Mrtfa*$^{-/-}$;*Mrtfb*$^{fl/fl}$ MEFs on were treated with 4OHT on day 0 and RNA collected for RNAseq analysis on day 0, 2, 4, and 12 (samples T0, T2, T4 and T12 respectively).

B) Gene Ontology (GO) analysis of 189 genes significantly up- or downregulated (>2-fold change, Padj <0.05) in *Mrtfa*$^{-/-}$*Mrtfb*$^{fl/fl}$ T0 pools compared with wildtype MEF pools. The top 10 GO terms (BP: Biological Process, MF: Molecular Function, CC: Cellular component) the Benjamini-Hochberg adjusted p-value for each set.

C) WT MEF pools 79, 80, 82; and *Mrtfa*$^{-/-}$*Mrtfb*$^{fl/fl}$ MEF pools 84, 86, 89 were treated or not with 4OHT and cultured for 0, 2, 4, or 12 days before RNAseq analysis (T0, T2, T4, T12 samples, see Figure 5A). Plot shows z-scores of genes showing a >2-fold change with Padj <0.05 for each pool in all samples, ordered according to unsupervised clustering of the T12 data. T0 and T12 data also shown in Figure 5A.

D) RT-qPCR analysis of adipogenic markers *Pparg*, *Cebpa*, and *Fabp4*. Data are mean of three independent experiments done in triplicate ± SEM. Statistical analysis by two-way ANOVA; $p<0.05$.

E) Oil Red O staining of WT or *Mrtfab*$^{-/-}$ MEFs cultured in regular or adipogenic differentiation medium (with 0.5mM IBMX, 1μM dexamethasone, 10μM insulin) for 20 days. Data are representative of two independent experiments.

Journal of Cell Science • Supplementary information

Figure S6

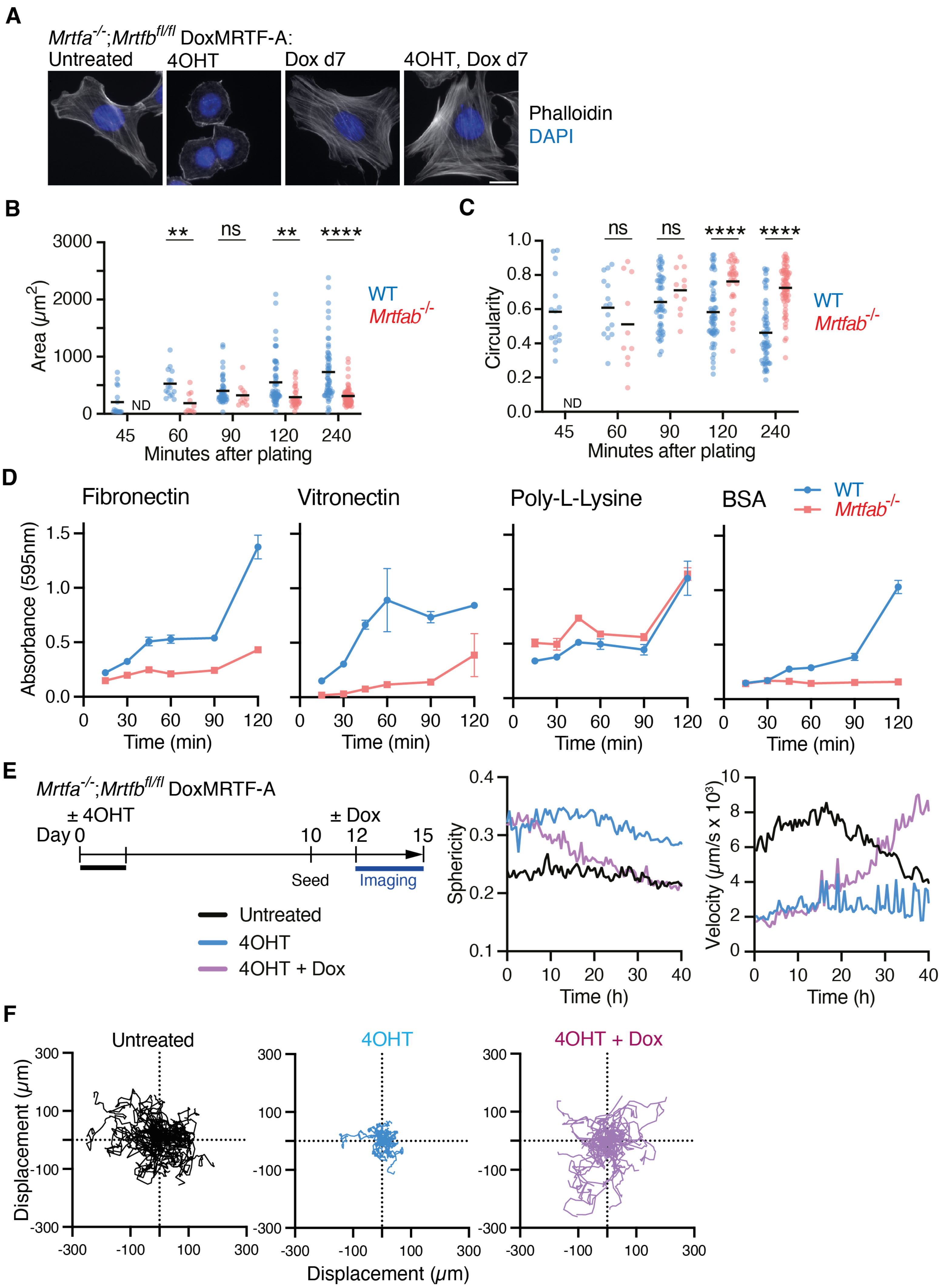

Journal of Cell Science • Supplementary information

Fig. S6 Cytoskeletal defects in *Mrtfab*$^{-/-}$ MEFs

A) Cell morphology at day 12 in line 1F2 *Mrtfab*$^{-/-}$ DoxMRTF-A cells treated as in Figure 3A. F-actin is stained using phalloidin. Images are representative images of 2 biological replicates (1F2 and 1F5 cell lines). Scale bar, 20µm.

B) WT or *Mrtfab*$^{-/-}$ MEFs (pools 80 and 86 respectively) were seeded and stained with CellMask Orange followed by fixation at indicated times after seeding. Cell areas were measured (>10 cells per timepoint); mean is indicated by the line. Statistical analysis, unpaired t-test; ND, not determined; ns, not significant; **, p<0.01; ****, p<0.0001.

C) Circularity of cells from (B). Analysis as in (B).

D) Adhesion assay on fibronectin, vitronectin, Poly-L-Lysine, or BSA. 50,000 cells pool 80 WT or pool 86 *Mrtfab*$^{-/-}$ MEFs were seeded per assay, stained with crystal violet at the indicated times, and adherent numbers quantified by absorbance at 595nm. Data are mean of three technical replicates ± SEM, and are representative of 5 independent experiments.

E) Protocol for phasefocus experiment. *Mrtfa*$^{-/-}$;*Mrtfb*$^{fl/fl}$ DoxMRTF-A cells (line 1F2) were treated or not with 4OHT and/or subsequently with doxycycline, and imaged for the next three days at 2 images / h (see Movies 3-8) and mean sphericity instantaneous velocity determined. Velocity of the untreated cells decreases at later times as cell migration becomes compromised by confluence. Note that upon Dox treatment GFP+ cells, re-expressing MRTF-A, accumulate slowly over a period of 10-15h in 4OHT-treated *Mrtfa*$^{-/-}$ *Mrtfb*$^{fl/fl}$ cells (see Movie 7,8). Similar results were observed with other lines.

F) Displacement plots of 1F2 cells treated as in (E). Cells (were monitored until division, exit of frame of view, or end of the video and 50 cell tracks plotted for each culture. Similar results were obtained with line 1F5 and 1B2.

Journal of Cell Science • Supplementary information

Figure S7

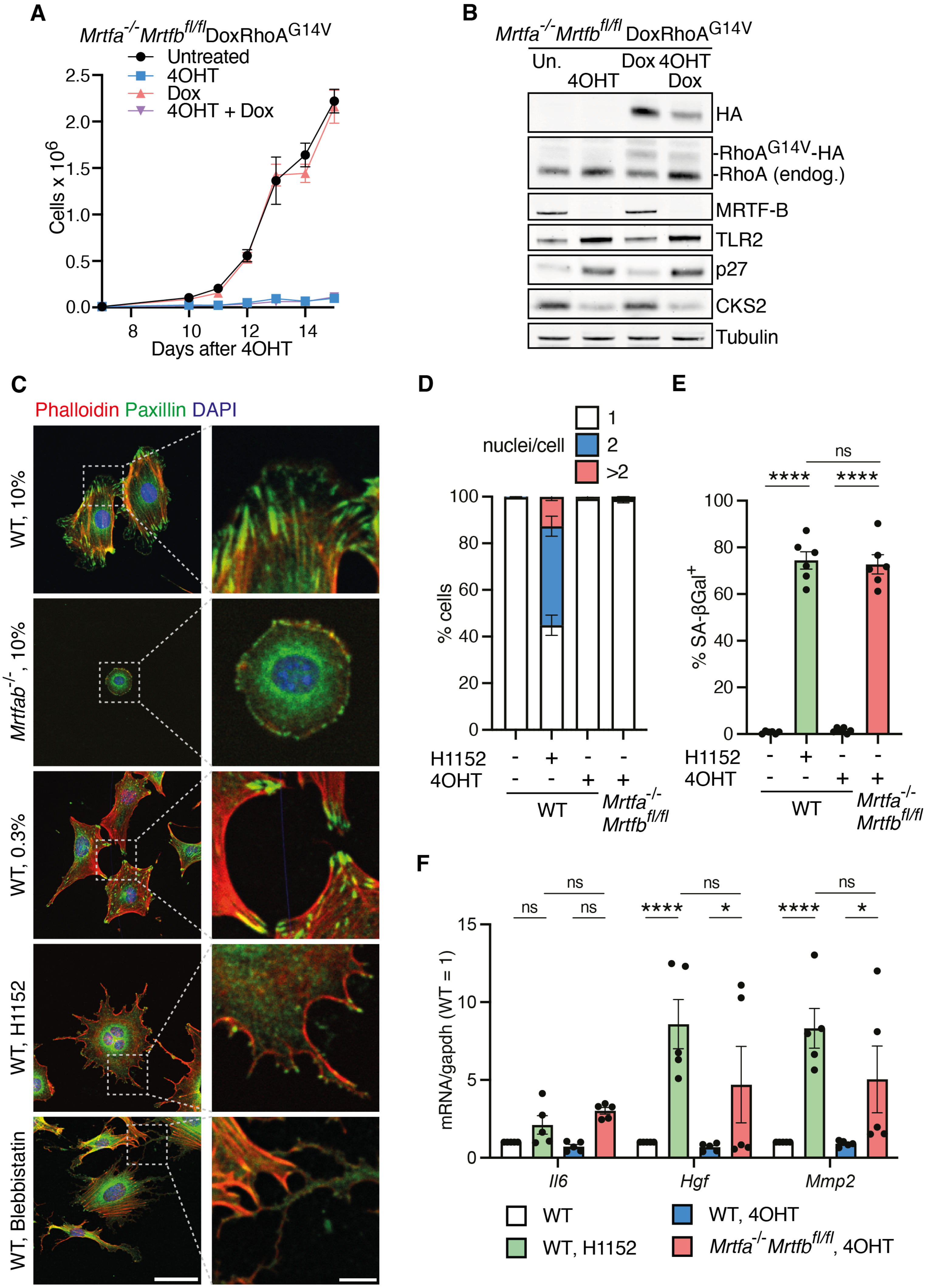

Journal of Cell Science • Supplementary information

Fig. S7. Inhibition of contractility in wildtype cells induces a phenoptype similar to MRTF inactivation

A) Pool 86 *Mrtfa*$^{-/-}$*Mrtfb*$^{fl/fl}$*Rosa26*$^{Tam\text{-}Cre}$ MEFs were infected with lentivirus pCW-RhoA^{G14V}-HA carrying dox-inducible constitutively active RhoA. Two days after plating, cells were treated or not with 4OHT and/or doxycycline. Cells were counted on day 10-15 after 4OHT. Data are mean ± SEM from 3 independent experiments.

B) Representative immunoblot of pool 86 *Mrtfa*$^{-/-}$*Mrtfb*$^{fl/fl}$DoxRhoA^{G14V} cells from (A) on day 12 after 4OHT treatment.

C) Immunofluorescence staining of paxillin (focal adhesions), phalloidin (F-actin) and DAPI (DNA) in pool 80 WT or pool 86 *Mrtfab*$^{-/-}$ MEFs treated as in Figure 7D. Scale bar = 50µm (left) and 10µm (right). Representative of 3 independent experiments.

D) Nuclear counts in pool 80 WT or pool 86 *Mrtfab*$^{-/-}$ MEFs on day 12 after 4OHT and/or H1152 treatment as in Figure 7D. >10 cells analysed per replicate sample Data mean ± SEM of 6 replicates.

E) SA-βgal staining of pool 80 WT or pool 86 *Mrtfab*$^{-/-}$ MEFs on day 12 after 4OHT and/or H1152 treatment as in Figure 7D. Data are mean ± SEM of three individual experiments, >100 cells scored per sample, n=3; significance, unpaired t-test; ns, not significant; ****, p<0.0001.

F) RT-qPCR of mRNA levels of SASP factors in pool 80 WT or pool 86 *Mrtfab*$^{-/-}$ MEFs on day 12 after 4OHT and/or H1152 treatment as in Figure 7D. Data are mean of 5 independent experiments ± SEM; significance, unpaired t-test; ns, not significant; *, p<0.05; ****, p<0.0001.

Journal of Cell Science • Supplementary information

Table S1. Differential gene expression upon MRTF inactivation in MEFs

WT or *Mrtfa*$^{-/-}$;*Mrtfb*$^{fl/fl}$ MEFs were treated with 4OHT on day 0, and RNA collected for RNAseq analysis on day 0, 2, 4, and 12. Differential gene expression analysis was carried out (samples T0, T2, T4 and T12 respectively). Each table presents differential gene expression analyses between WT and *Mrtfa*$^{-/-}$*Mrtfb*$^{fl/fl}$ (MRTFdKO) MEFs at each time point, ranked from maximum fold-downregulation to maximum fold-upregulation in MRTFdKO MEFs compared with wildtype MEFs. Columns are: ENSEMBL, ensemble gene ID; baseMean, average normalised expression across samples; log2FoldChange, $\log_2$-transformed fold change between conditions; lfcSE, standard error of the $\log_2$ fold change; pvalue, raw p-value from Wald test; padj, p-value adjusted for multiple testing using Benjamini-Hochberg (BH); SYMBOL, gene symbol; ENTREZID, Entrez gene ID; -log10(pvalue), negative $\log_{10}$-transformed adjusted p-value for volcano plots.

Available for download at
https://journals.biologists.com/jcs/article-lookup/doi/10.1242/jcs.264444#supplementary-data

Journal of Cell Science • Supplementary information

Table S2. GSEA and GO analysis of genes downregulated at day 0 or 12 after MRTF-inactivation

Sheet 1: Gene Set Enrichment Analysis (GSEA) table at T12 showing significant gene sets of the Hallmark category. Analysed in R using GSEA() from clusterprofiler with Hallmark gene sets from msigdbr. Columns are: ID, Hallmark identifier of gene set; setSize, number of genes from the gene set present in the data; enrichmentScore, running-sum statistic of degree of enrichment; NES, Normalised Enrichment Score – enrichment score normalised by gene set; pvalue, raw p-value from permutation test of enrichment; p.adjust, p-value adjusted for multiple comparisons using Benjamini-Hochberg (BH); qvalue, estimated false discovery rate (FDR); rank, position of peak enrichment score in the ranked gene list; leading_edge, summary of genes driving enrichment; core_enrichment, gene symbols in leading edge subset.

Sheet 2: Gene Ontology (GO) analysis table at T12 showing significant upregulated gene ontology gene lists. Analysed using enrichGO() from clusterprofiler with ont = "ALL". Columns are: ONTOLOGY, category; ID, GO term identifier; Description, name of gene ontology term; GeneRatio, proportion of input genes in the GO term; BgRatio, proportion of background genes in the GO term; pvalue, raw p-value from hypergeometric test; p.adjust, p-value adjusted for multiple comparisons using Benjamini-Hochberg (BH); qvalue, estimated false discovery rate (FDR); geneID, input genes annotated to the GO term; geneSYMBOL, input genes annotated to the GO term; Count, number of input genes annotated to the GO term.

Sheet 3: As sheet 2, but for significantly downregulated genes at T12.

Sheet 4: As sheet 2, but for significantly upregulated genes at T0.

Sheet 5: As sheet 2, but for significantly downregulated genes at T0.

Available for download at
https://journals.biologists.com/jcs/article-lookup/doi/10.1242/jcs.264444#supplementary-data

Table S3. Oligonucleotides and guides

Available for download at
https://journals.biologists.com/jcs/article-lookup/doi/10.1242/jcs.264444#supplementary-data

Table S4. Antibodies.

Available for download at
https://journals.biologists.com/jcs/article-lookup/doi/10.1242/jcs.264444#supplementary-data

Journal of Cell Science • Supplementary information

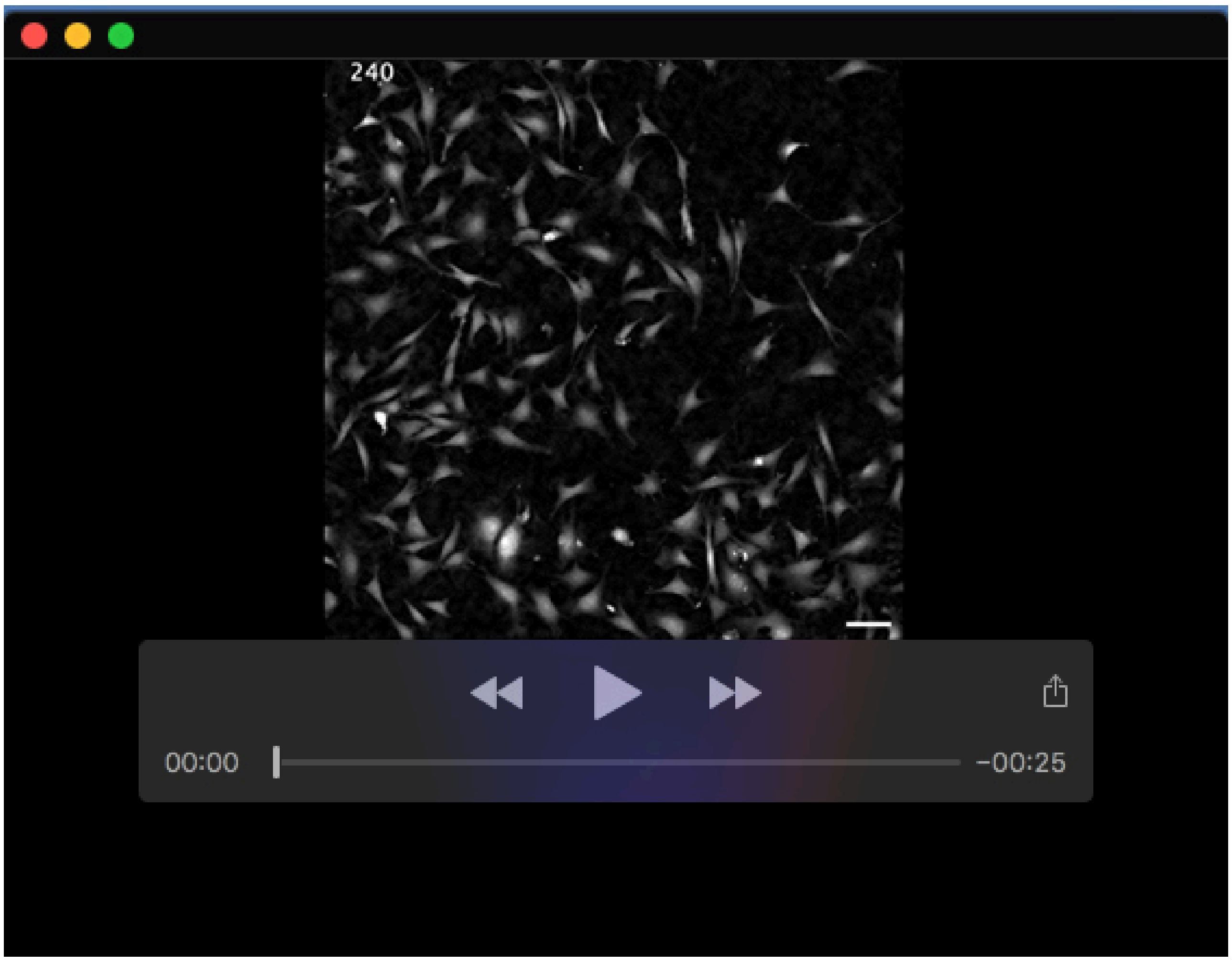

Movie 1. Timelapse microscopy of wildtype and *Mrtfab*$^{-/-}$ MEFs. Cells were filmed over a 96h period, with 1 frame per 30 minutes.

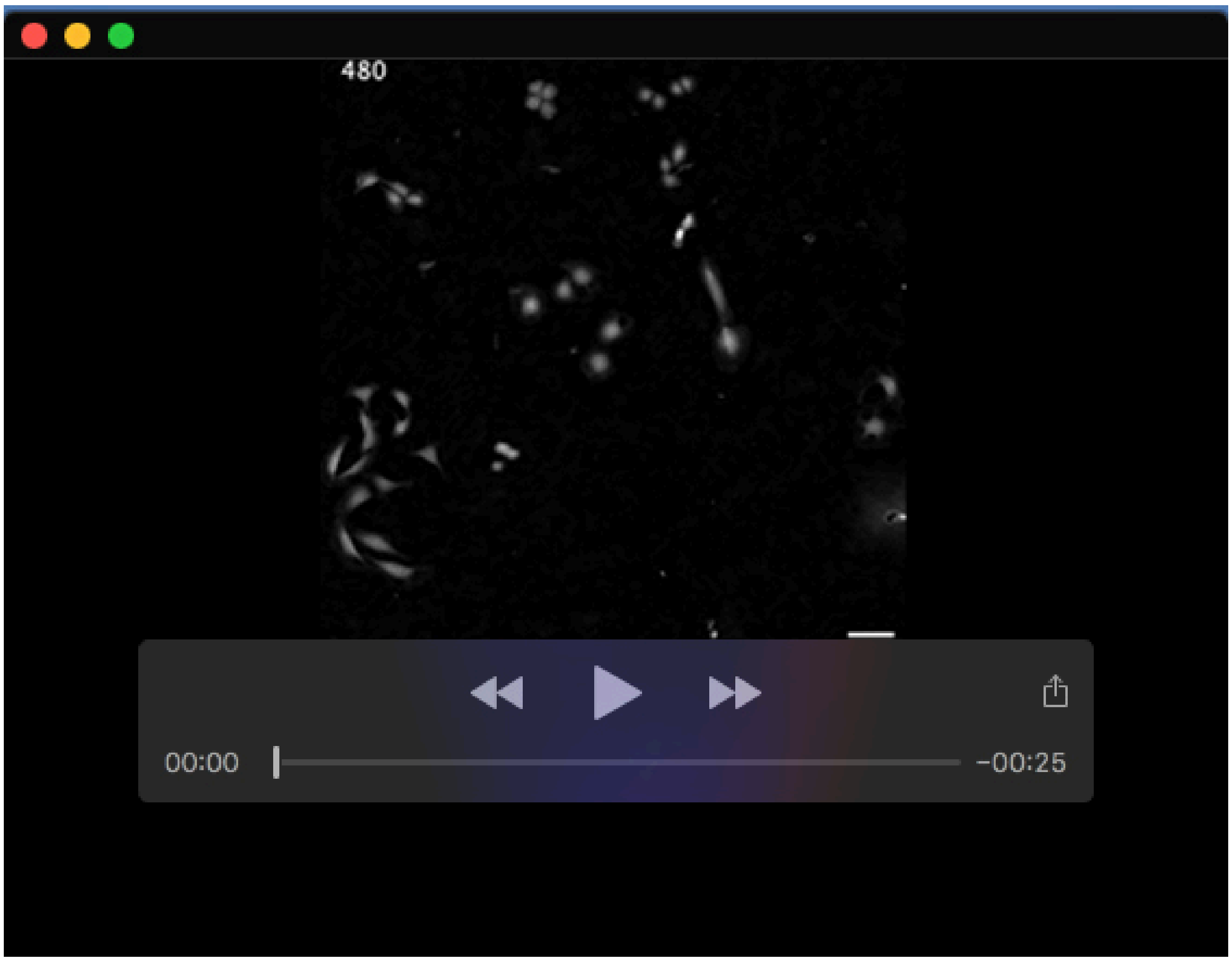

Movie 2. Timelapse microscopy of wildtype and *Mrtfab*$^{-/-}$ MEFs. Cells were filmed over a 96h period, with 1 frame per 30 minutes.

Journal of Cell Science • Supplementary information

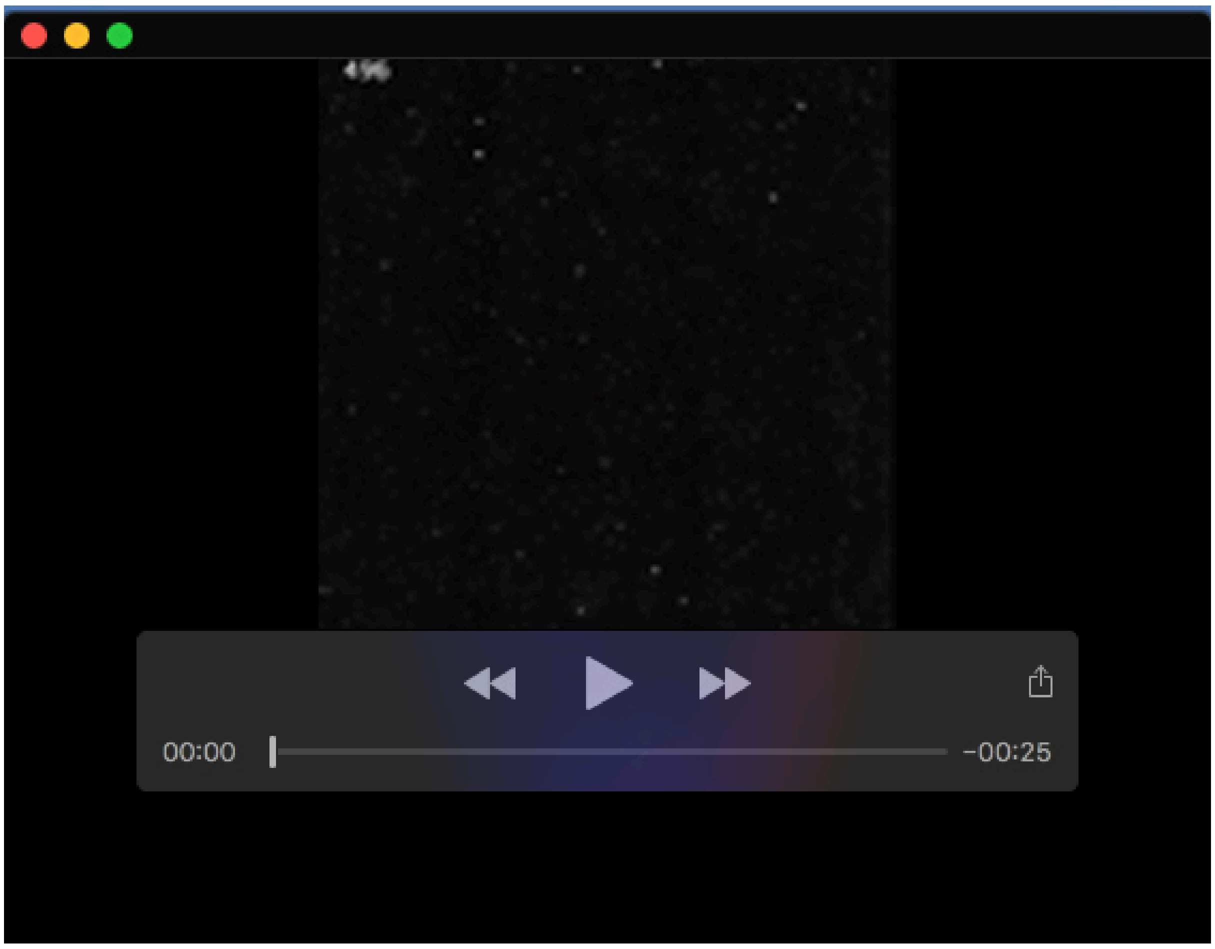

Movie 3. MRTF-A reexpression restores morphology, motility, and proliferation. *Mrtfa*$^{-/-}$*Mrtfb*$^{fl/fl}$ DoxMRTF-A cells were treated as in Fig. 5E and analysed by Phasefocus livecyte microscopy at day 12 for 40h at 2 images/h. GFP images. Untreated cells.

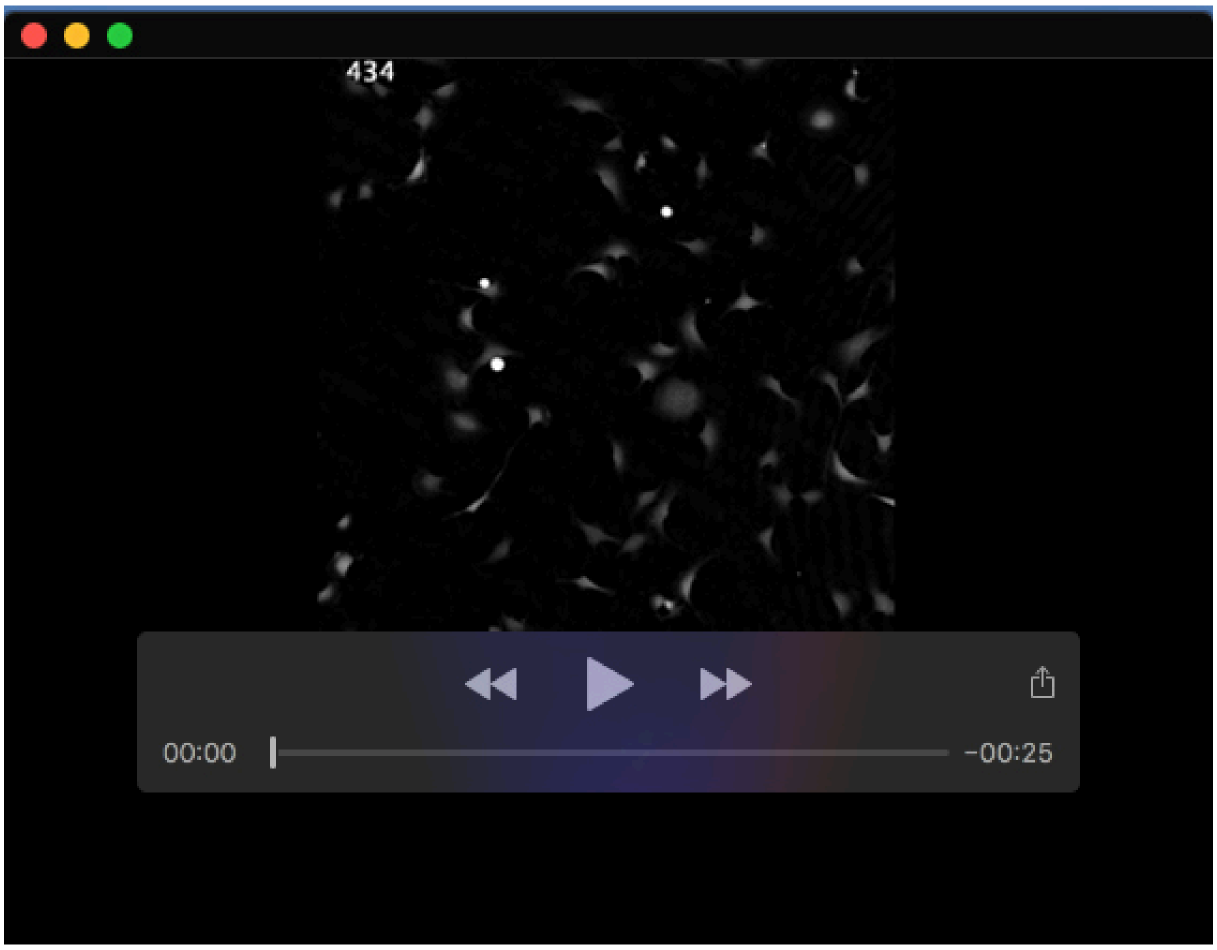

Movie 4. MRTF-A reexpression restores morphology, motility, and proliferation. *Mrtfa*$^{-/-}$*Mrtfb* DoxMRTF-A cells were treated as in Fig. 5E and analysed by Phasefocus livecyte microscopy at day 12 for 40h at 2 images/h. Phase contrast images. Untreated cells.

Journal of Cell Science • Supplementary information

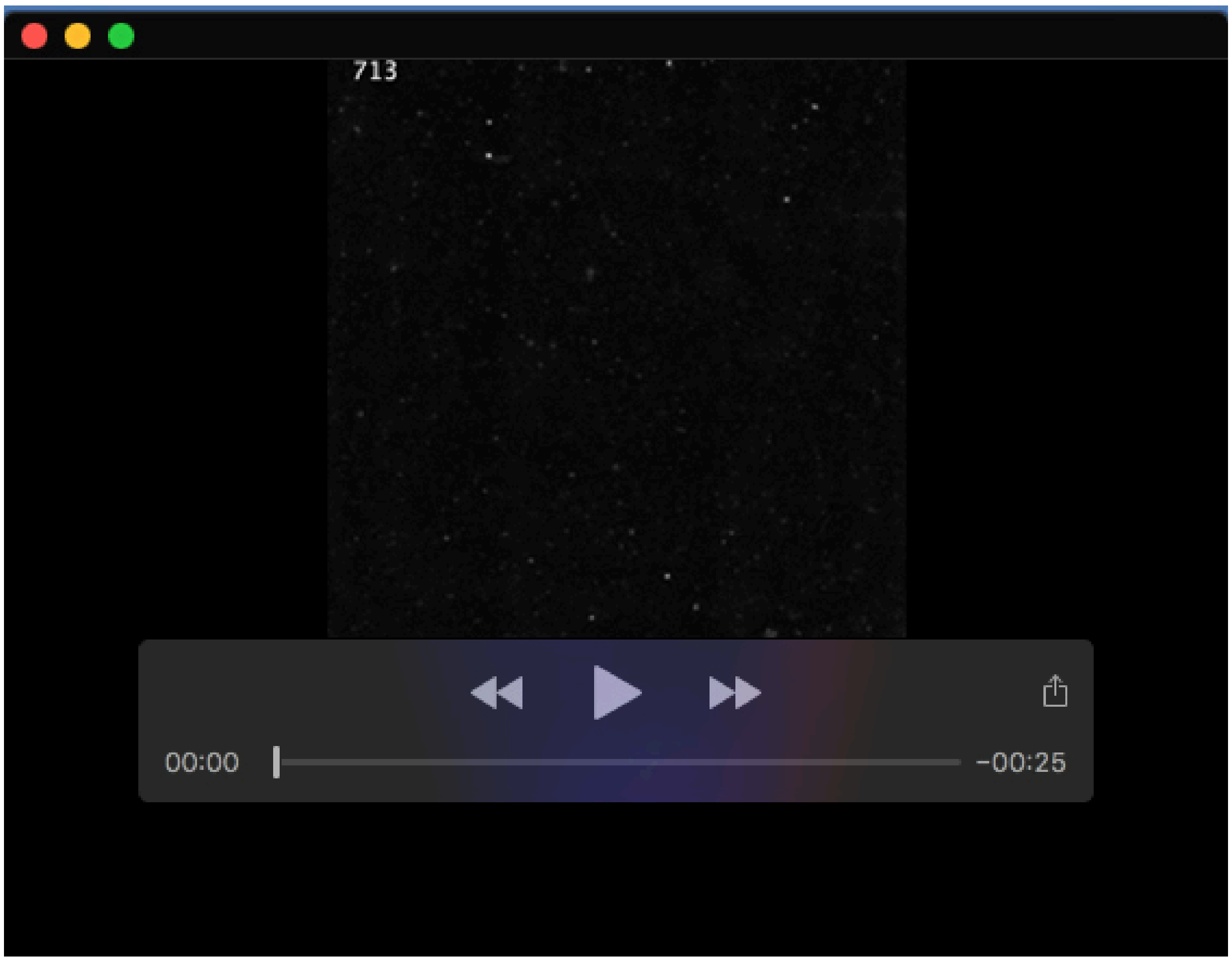

Movie 5. MRTF-A reexpression restores morphology, motility, and proliferation. *Mrtfa*$^{-/-}$*Mrtfb*$^{fl/fl}$ DoxMRTF-A cells were treated as in Fig. 5E and analysed by Phasefocus livecyte microscopy at day 12 for 40h at 2 images/h. GFP images. Cells treated with 4OHT.

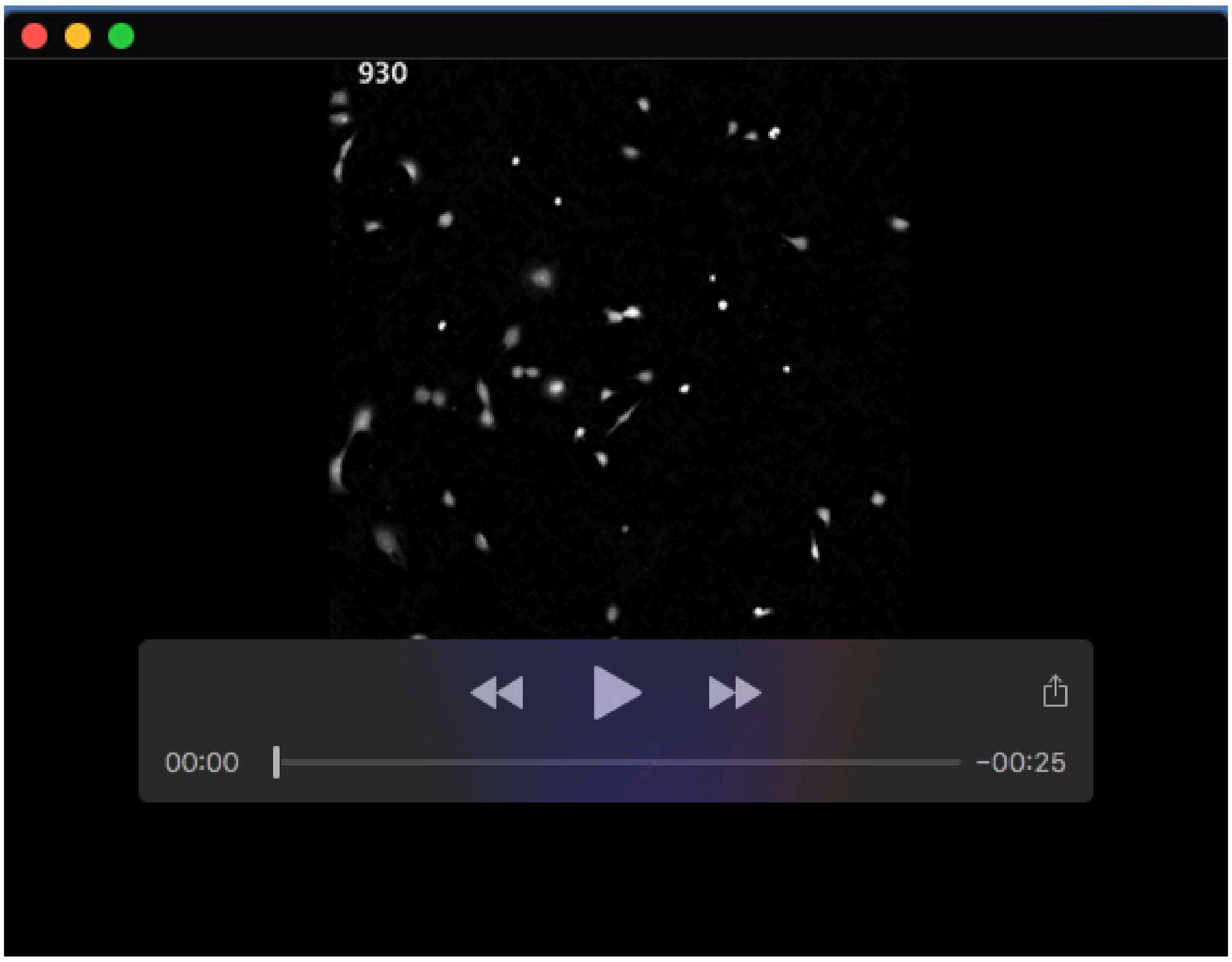

Fig. 6. MRTF-A reexpression restores morphology, motility, and proliferation. *Mrtfa*$^{-/-}$*Mrtfb*$^{fl/fl}$ DoxMRTF-A cells were treated as in Fig. 5E and analysed by Phasefocus livecyte microscopy at day 12 for 40h at 2 images/h. Phase contrast images. Cells treated with 4OHT.

Journal of Cell Science • Supplementary information

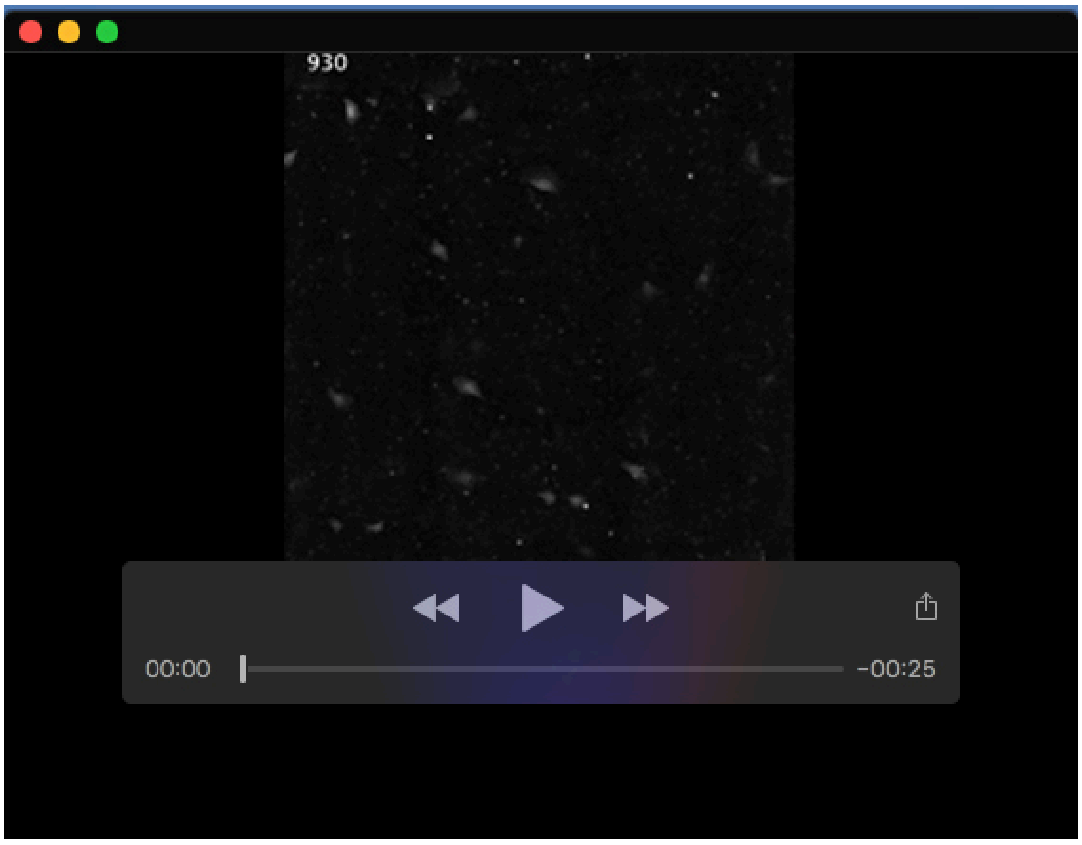

Movie 7. MRTF-A reexpression restores morphology, motility, and proliferation. *Mrtfa*$^{-/-}$*Mrtfb*$^{fl/fl}$ DoxMRTF-A cells were treated as in Fig. 5E and analysed by Phasefocus livecyte microscopy at day 12 for 40h at 2 images/h. GFP images. 4OHT-treated cells at day 12 treated with Dox 1h prior to imaging. Velocity of the untreated cells decreases at later times as cells become confluent, which was not reached by the Dox-treated cells (compare Movies 3,5). Note that upon Dox treatment GFP+ cells, re-expressing MRTF-A, accumulate slowly over a period of 10-15h in 4OHT-treated *Mrtfa*$^{-/-}$*Mrtfb*$^{fl/fl}$ cells.

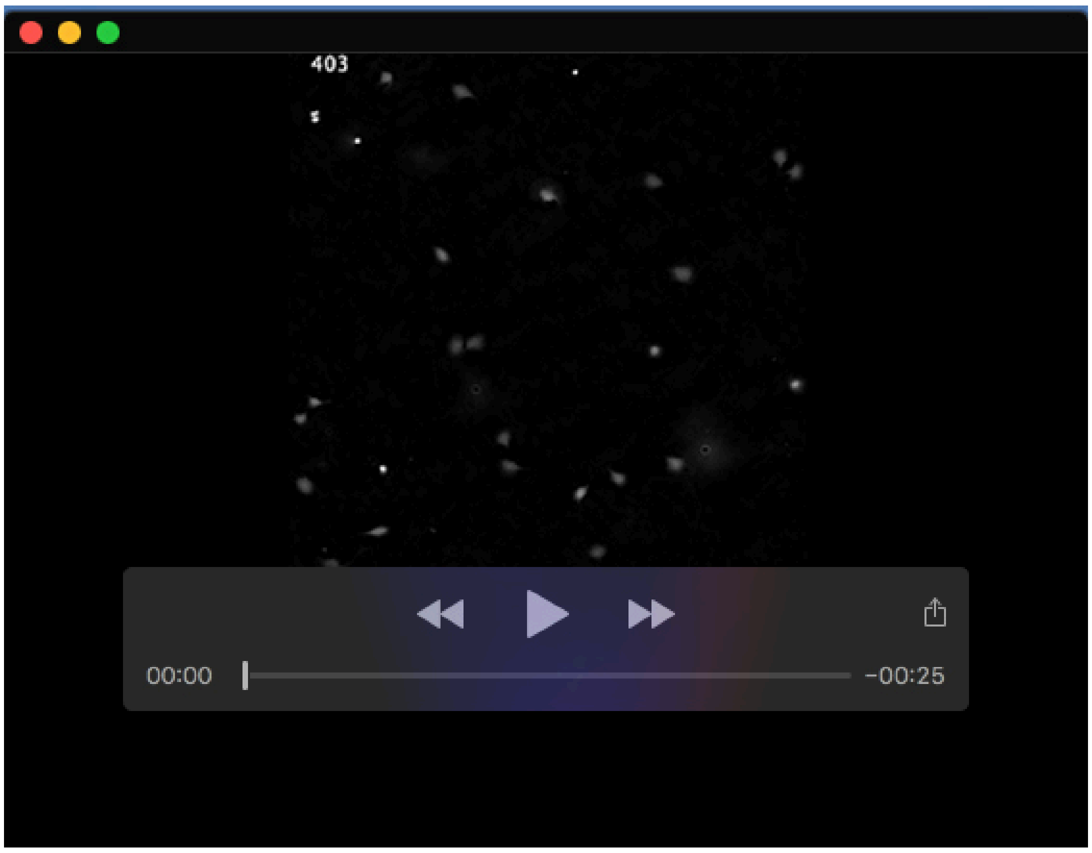

Movie 8. MRTF-A reexpression restores morphology, motility, and proliferation. *Mrtfa*$^{-/-}$*Mrtfb*$^{fl/fl}$ DoxMRTF-A cells were treated as in Fig. 5E and analysed by Phasefocus livecyte microscopy at day 12 for 40h at 2 images/h. Phase contrast images. 4OHT-treated cells at day 12 treated with Dox 1h prior to imaging. Velocity of the untreated cells decreases at later times as cells become confluent, which was not reached by the Dox-treated cells (compare Movies 3,5).

Journal of Cell Science • Supplementary information
